# Transcultural adaptation and validation of the Serbian version of Functional Assessment of Chronic Illness Therapy—Treatment Satisfaction—Patient Satisfaction (FACIT-TS-PS) questionnaire

**DOI:** 10.1371/journal.pone.0294339

**Published:** 2023-11-15

**Authors:** Ivana Radovic, Igor Krdzic, Ana Jovanovic, Danka Vukasinovic, Ivan Soldatovic, Masa Petrovic, Ana Tomic, Tanja Jesic-Petrovic, Aleksandar Matejic, Bojana Salovic, Ilic-Zivojinovic Jelena

**Affiliations:** 1 Department of Pretransfusion Testing, Blood and Blood Products Issuing and Heamovigilance, Institute for Blood Transfusion of Serbia, Belgrade, Serbia; 2 Department of Colorectal Surgery, University Clinical Hospital Center Zvezdara, Surgery Clinic “Nikola Spasić”, Belgrade, Serbia; 3 Faculty of Medicine, Institute of Hygiene and Medical Ecology, University of Belgrade, Belgrade, Serbia; 4 Faculty of Medicine, Institute of Medical Statistics and Informatic, University of Belgrade, Belgrade, Serbia; 5 Faculty of Medicine, University of Belgrade, Belgrade, Serbia; 6 Center of Excellence, Institute for cardiovascular diseases “Dedinje”, Belgrade, Serbia; 7 Public Health Care Center Doboj, Doboj, Bosnia and Herzegovina; 8 Department of plastic and reconstructive surgery, Institute for Orthopedic Surgery “Banjica”, Belgrade, Serbia; Nova Southeastern University / Yarmouk University, UNITED STATES

## Abstract

**Objective:**

Transcultural adaptation and validation of FACIT-TS-PS questionnaire to Serbian language.

**Methods:**

Standard forward and backward translation from English to Serbian language was performed. Pilot testing of FACIT-TS-PS was conducted on 12 patients with a confirmed diagnosis of malignant tumor. The study included 154 patients with malignant disease. The Questionnaire of Patient Satisfaction was used as a validated tool to evaluate concurrent validity of FACIT-TS-PS questionnaire. Reproducibility was tested on 30 subjects who answered the questionnaire for the second time two weeks later.

**Results:**

Three FACIT-TS-PS subscales (Physician Communication, Treatment Staff Communication and Nurse Communication) demonstrated satisfactory construct validity using Cronbach’s alpha, the remaining two subscales (Technical Competence and Confidence & Trust) showed high ceiling effect. Treatment Staff Communication subscale showed large floor effect. Concurrent validity was demonstrated by correlation with the two dimensions of the Questionnaire of Patient Satisfaction. Satisfactory reproducibility was demonstrated on 30 patients who filled the questionnaire for the second time two weeks after initial interview.

**Conclusion:**

The Serbian version of FACIT-TS-PS with the omission of Treatment Staff Communication subscale could be used as a valid instrument to assess patient and treatment satisfaction in chronically ill patients in the Serbian population. Omission of Treatment Staff Communication subscale is necessary because it contains questions not relevant for patients in Serbian healthcare system.

## Introduction

Patient ratings of health care are an integral component of patient-centered care [1]. There are two major types of patient-reported health care ratings: patient satisfaction and patient reports of their actual experiences. Patient satisfaction includes patient concerns about their disease and its treatment, issues of treatment affordability and financial burden for the patient, communication with health care providers, access to services, satisfaction with treatment explanations, and confidence in their physician. Patient reports of their actual experiences with health care services are often regarded as more specific, actionable, understandable, and objective compared to general ratings alone [2].

It is often very difficult to completely understand a patient satisfaction regarding health services. Both care-related and non-service factors play a significant role in users’ health care perception. Elements linked to care itself combined with those unrelated to the provision of services can have a substantial impact on how people perceive their overall experience of receiving medical care, even when the treatment’s outcome is sufficient [3, 4].

The most extensively studied possible indicators of patient satisfaction are healthcare service-quality factors like medical practitioner’s competence, their experience and education, and treatment outcomes, which have a significant and mostly beneficial effect [5–7]. Non-service factors, such as political support and cultural change, can play into patient satisfaction with changes to improve the quality of medical services [8].

Patient-related factors like education and overall health status can also have an impact on patient satisfaction scores, [9] but even the influence of these variables can be modified with certain medical practice determinants, such as good physician-patient communication, interpersonal skills and meeting the patient’s pre-visit expectations [10, 11].

The assessment of satisfaction has evolved into a continuous process that periodically informs physicians and hospital management how health service is being delivered and if patient expectations are being met and fulfilled [12]. This highlights the importance of research on which aspects and determinants of health care have the greatest impact on patient satisfaction that can potentially contribute to public health policymaking [13].

From a clinician’s perspective, evaluating what makes patients satisfied with health institutions and services is very important as well, as it is also relates to the extent to which patients comply with the recommended medical intervention or treatment [14]. Furthermore, satisfied patients tend to take a more active role in their treatment and are more likely to continue treatment in a certain institution, keep the same health insurance provider, and are more accepting of the healthcare system in general [14–16]. In this way, patient satisfaction could increase survival rates in certain groups of patients, primarily those suffering from cancer, but also other chronic diseases that require long-term treatment [17].

The aim of this study is to validate Functional Assessment of Chronic Illness Therapy—Treatment satisfaction—Patient satisfaction (FACIT-TS-PS) measure [18, 19]. This questionnaire is a part of the FACIT system, a collection of measures and symptom indicators primarily focused on cancer and other chronic diseases. The FACIT system initially began measuring the Functional Assessment of Cancer Treatment (FACT-G) and since then, additional scales and indicators to cover other conditions and treatment-related aspects have been added. Each FACIT scale or index is specific enough for clinically relevant issues but still allows comparisons between patients with different chronic illnesses if needed (available at www.facit.org).

## Method

### Questionnaires

FACIT-TS-PS is a self-administered questionnaire developed and validated by Peipert et al. [18] which measures multiple aspects of patient satisfaction including communication, trust and overall ratings. It is currently available in 7 different languages and validated in the US and Mexican patient population [18, 19]. Both countries have very distinct healthcare systems. The Mexican public healthcare system is fully or partially subsidized by the federal government, bearing a strong resemblance to the Serbian healthcare system [20]. Contrarily, the US healthcare is mainly provided by private sector healthcare facilities and paid for by a combination of public programs, private insurance and out-of-pocket payments [21]. In addition, both Mexico and Serbia are considered as upper-middle-income economies, contrary to the US [22]. The FACIT-TS-PS consists of 26 items organized in 5 domains: Physician Communication PC (12 items), Treatment Staff Communication TSC (4 items), Technical Competence TC (3 items), Nurse Communication NC (3 items), Confidence & Trust CT (4 items), and 3 questions treated as individual items which are not included in any summary scores. The questionnaire is designed to be completed by patients 18 years and older with a chronic illness who are currently undergoing treatment. The questionnaire aims to evaluate their satisfaction in the last seven days. The 26 items are scored on a 4 point Likert scale (0 = No, not at all, 1 = Yes, but not as much as I wanted, 2 = Yes, almost as much as I wanted, 3 = Yes, and as much as I wanted). Of the three individual items, two items are scored on 3 point Likert scale (0 = No, 1 = Maybe, 2 = Yes) and the third individual item is scored on 5 point Likert scale 5 (0 = Poor, 1 = Fair, 3 = Good, 4 = Very Good, 5 = Excellent). Domain scores are calculated as the sum of all individual items’ scores, multiplied by the number of items in the domain, and divided by the number of the items answered [23].

For an assessment of patients’ satisfaction the Questionnaire of Patient Satisfaction (QPS) is widely used in Serbia. QPS is a 20 item questionnaire that was designed by the Institute of Public Health of Republic of Serbia “Dr Milan Jovanovic Batut” and validated by Vuković et al. [16]. The questionnaire is composed of 5 items assessing demographic and socio-economic indicators, 3 items regarding information on whether the patient has a personal GP, the frequency of visits to a GP, and the place where the patient receives advice from nurses regarding healthy lifestyles, and 12 items that aim to evaluate patient satisfaction. Ten of the items are scored on a 3 point Likert scales, and two items on a 5-point Likert scale. The QPS evaluates two domains: patient satisfaction with medical staff and contextual patient dissatisfaction. The scores for each domain is calculated as the sum of the number of points for each individual item multiplied by the given component loading for that item [16].

### Translation and cultural adaptation

The translation of FACIT-TS-PS was conducted following the standard FACIT translation methodology and internationally accepted methodology for cross-cultural adaptation and validation of questionnaires. The forward translation from English into Serbian was performed by two translators, working independently from one another. The third translator reviewed the English version and two translations into the Serbian language. A fourth translator performed a back translation from Serbian into English. Then, the back translation was reviewed by the translation project team. A team of clinical experts provided feedback on the acceptability of the forward and back translation. All versions of the questionnaire were submitted to the licence holder.

### Pilot testing

Pilot testing of the pre-final Serbian version of the questionnaire was performed in February 2019 on 12 randomly selected patients with a confirmed diagnosis of malignant disease, who were Serbian native speakers, able to read and write and give verbally informed consent. Patients completed the FACIT-TS-PS and the Patient Interview form prepared by FACIT organization. Patients were asked to read all of the questions, even those that did not seem relevant, in order to evaluate the wording of the questions and statements. None of the patients found the questions difficult to understand, offensive, irrelevant, disturbing or upsetting. No major challenges occurred during the pilot testing. The data from pilot testing were submitted and approval was obtained from FACIT-TS-PS questionnaire licence holder.

### Study subjects

The study was conducted from September 1^st^ 2019 until Jun 30^th^ 2020 at the University Clinic for Surgery “Nikola Spasić” of Clinical Center Zvezdara, in Belgrade, Serbia. During the period from April 1st to May 29th 2020 the Clinical Center Zvezdara functioned as a COVID hospital, and did not accept any other patients. During this period patient recruitment and data collection were temporally suspended. It included 154 patients, recruited by availability that were hospitalized or were treated in the outpatient setting. Inclusion criteria were age between 18 and 70, diagnosis of any type of cancer regardless of a stage and previous or current treatment modality, and more than 6 months of life expectancy. All subjects were Serbian native speakers and were able to communicate. Exclusion criteria were presence of cognitive impairment or psychosis. The patients completed the questionnaires by themselves and for those who had visual, literal, or some other impairment, a trained interviewer was provided.

All the patients signed a written consent form. The study was approved by the Ethics Committee of the Medical Faculty University of Belgrade (No 2650/X-16).

### Construct validity

Internal consistency, measured by Cronbach’s alpha coefficient [24], evaluates if all domains of an instrument measure the same construct. Values above 0.60 were considered satisfactory, and above 0.70 ideal.

Concurrent validity was tested by correlating the FACIT-TS-PS subscales and total score with both dimensions of the QPS. Spearman correlation coefficient was used.

### Reliability

Thirty subjects filled the FACIT-TS-PS questionnaire for a second time two weeks after the initial interview. We explored test-retest reliability by Related-Samples Wilcoxon Signed Rank Test and Pearson correlation coefficient. Interpretation of values were as follows: 0.90–1.00 a very strong correlation, 0.70–0.89 a strong correlation, 0.40–0.69 a moderate correlation, 0.10–0.39 a weak correlation and <0.10 a negligible correlation, p<0.05 was considered statistically significant [25].

### Data analysis

Results are presented as count (%), means±standard deviation or median (25^th^ Percentile-75^th^ Percentile) depending upon data type and distribution. Normality of distribution was assessed by Kolmogorov–Smirnov test and by visual analysis of histogram of frequencies and Q-Q plot. Data were analysed using the statistical package SPSS (Statistical Package for the Social Sciences) v.29.0 (IBM Corp., Armonk, USA).

## Results

### Socio-demographic and clinical characteristics of subjects

The main characteristics of the study sample are presented in Table 1. Approximately half of the subjects were male. On average, patients included were elderly (≥65 years old) and slightly overweight (BMI≥25). The majority of subjects were married and had completed primary or secondary school. Colorectal cancer was the most prevalent diagnosis and patients in all stages of disease were included. From the study population, more than half of patients had previously undergone a classic surgery and nearly a quarter had previously undergone laparoscopic surgery. Around 17% received radio and chemotherapy. Complications were rare. The majority of patients were hospitalized for more than 10 days, and a quarter was hospitalized during the study.

**Table 1:** Socio-demographic and clinical characteristics of study subjects.

	N (%)/ mean±sd
Male gender	85 (55.2)
Age (yrs.)	67.8±10.0
Body height (cm)	171.1±9.2
Body mass (kg)	74.9±14.9
BMI (kg/m2)	25.5±4.4
Marital status	
single	7 (4.5)
married	104 (67.5)
divorced	14 (9.1)
widowed	29 (18.8)
Education level	
primary	27 (17.5)
secondary	84 (54.5)
high/university	39 (25.3)
no formal education	4 (2.6)
Type of cancer	
duodenal cancer	1 (0.6)
hepatic cancer	3 (1.9)
colorectal cancer	131 (85.1)
pancreatic cancer	5 (3.2)
gastric cancer	11 (7.1)
gallbladder cancer	3 (1.9)
Stage of cancer	
I	18 (32.1)
II	16 (28.6)
III	14 (25)
IV	8 (14.3)
Type of treatment	
classic surgery	85 (55.2)
laparoscopic surgery	36 (23.4)
no surgery	33 (21.4)
Radiotherapy and chemotherapy before	3 (1.9)
Radiotherapy and chemotherapy after	23 (14.9)
Complication	13 (8.4)
Hospitalization	38 (24.7)
Duration of hospitalization	
<5 days	25 (16.2)
5–10 days	60 (39)
>10 days	69 (44.8)

### Descriptive statistics of FACIT-TS-PS

Measures of central tendency and dispersion, internal consistency, floor and ceiling effects for subscales of FACIT-TS-PS are displayed in Table 2. Internal consistency expressed by Cronbach’s Alpha is high in subscales PC and TSC and adequate in subscale NC. Internal consistency for TC and CT subscales is low. High ceiling effect is present in this two scales, also. High floor effect is observed in TSC subscale. Three items, which are not included in any summary scores, had the following median, 25^th^ percentile and 75^th^ percentile: TS38 Would you recommend this clinic or office to others? 2 (2–2), TS39 Would you choose this clinic or office again? 2 (2–2) TS40 How do you rate the care you received? 4 (4–4). No subject had minimum score for aforementioned questions, and percentages of subjects with maximum score were 98.7%, 98.1% and 74.7% respectively.

**Table 2:** Descripitve statistics, celling and floor effects of FACIT-TS-PS.

	Median (25^th^ Perc-75^th^ Perc)	item average, min, max	Cronbach’s Alpha	Cronbach’s Alpha del interval	Floor effect (%)	Ceiling effect (%)
PC	31 (26–34)	28.76 (6–36)	0.860	0.835–0.870	0	19.5
TSC	0 (0–4)	2.58 (0–12)	0.921	0.885–0.906	62.3	9.1
TC	9 (9–9)	8.78 (6–9)	0.230	0.146–0.211	0	87.7
NC	9 (7–9)	7.61 (0–9)	0.728	0.568–0.780	3.2	55.8
CT	12 (12–12)	11.63 (7–12)	0.372	0.224–0.348	0	78.6
FACIT-TS-PS total score	61 (54–66)	59.36 (29–78)			0	2.6

PC- Physician Communication, TSC- Treatment Staff Communication, TC- Technical Competence, NC- Nurse Communication, CT- Confidence and Trust

### Construct validity

We examined the influence of item deletion on internal consistency (Table 2). In the PC subscale, if items “TS11 Did your doctor(s) explain the possible side effects or risks of your treatment?” and “TS28 Did you have enough time to make decisions about your health care?” are omitted higher internal consistency can be achieved (0.864 and 0.870). Similar results were observed in the NC subscale, if item “TS32 Did your nurse(s) show genuine concern for you?” was deleted Cronbach’s alpha would rise to 0.780. In contrast, the deletion of items would not raise internal consistency of the TC and CT subscales.

### Concurrent validity

Concurrent validity was tested by Spearman correlation coefficient between subscales and total score of FACIT-TS-PS and two dimensions of the QPS (Table 3.). Scores obtained in the PC and CT subscales as well as the FACIT-TS-PS total score correlated positively, weakly with dimension 1, patient satisfaction with medical staff and negatively with dimension 2, contextual patient dissatisfaction. No correlation between TSC, TC and NC sub scores and the QPS were observed.

**Table 3:** Concurrent validity (Spearman correlation coefficient) of FACIT-TS-PS.

	PC	TSC	TC	NC	CT	FACIT-TS-PS total score
Dimension 1, patient satisfaction with medical staff	.167* 0.041	0.020	0.132	0.133	.196* 0.016	.161* 0.048
Dimension 2, contextual patient dissatisfaction	-.170* 0.038	-0.019	-0.139	-0.095	-.198* 0.015	-.164* 0.045

PC- Physician Communication, TSC- Treatment Staff Communication, TC- Technical Competence, NC- Nurse Communication, CT- Confidence and Trust

* p<0.05

### Reproducibility

Reproducibility of the FACIT-TS-PS questionnaire was tested on 30 subjects who answered the questionnaire for the second time two weeks later. Median, 25^th^ percentile and 75^th^ percentile of subscales’ and total score for test and retest are presented in Table 4. No significant difference between scores was confirmed by Related-Samples Wilcoxon Signed Rank Test. The reproducibility was high for PC, TSC, TC subscale and total score, which showed strong (TSC and total score) and moderate (PC and TC) highly statistically important linear correlation of test-retest results and good for NC subscale (moderate statistically important linear correlation). Only CT subscale presented with below expected reproducibility (r = 0.124, p = 0.515).

**Table 4:** Reproducibility of FACIT-TS-PS subscales.

FACIT-TS-PS subscales	test score	retest score	p^a^	r ^b^
PC	33 (31–36)	33 (29–36)	0.755	0.637**
TSC	1 (0–7)	2 (0–10)	0.721	0.806**
TC	9 (9–9)	9 (9–9)	0.317	0.695**
NC	9 (7–9)	9 (7–9)	0.552	0.406*
CT	12 (12–12)	12 (12–12)	0.589	0.124
FACIT-TS-PS total score	63 (57–72)	66 (57–72)	0.684	0.799**

^a^ Related-Samples Wilcoxon Signed Rank Test

^b^ Pearson correlation coefficient

** p<0.01

* p<0.05

PC- Physician Communication, TSC- Treatment Staff Communication, TC- Technical Competence, NC- Nurse Communication, CT- Confidence and Trust

## Discussion

According to the data published by the Institute of Public Health of Serbia “Dr Milan Jovanovic Batut”, malignant diseases were the second leading cause of death before the start of the COVID-19 pandemic [26]. The annual number of newly diagnosed malignant cases was 41419 for 2020, 22110 (53.4%) of whom were male. The majority of newly diagnosed cases belonged to the elderly group, 63.3% and 54.7%, males and females above the age of 65, respectively [27]. Our study sample had similar gender and age distribution; however there is a bias toward colorectal cancer in our sample. In the Serbian population, the colon and rectum are the second leading site of the newly diagnosed cancer cases [27].

Internal consistency of the FACIT-TS-PS was good for PC and TSC subscales and acceptable for NC subscale. On the other hand, TC and CT subscales had very low internal consistency. The English version of FACIT-TS-PS showed adequate internal consistency between 0.72–0.95 [18]. The Arabic version of FACIT-TS-PS had high internal consistency for all subscales (0.854–0.966), except TSC (0.499) [28]. TC and CT subscales in our study had a very prominent ceiling effect, and both had a floor effect of zero. This may be a consequence of cultural influence. Our subjects were elderly and three quarters of them had completed secondary education or less. This population group, in Serbian society, usually has absolute trust in their physician and seldom questions physician’s decisions. Better adherence to prescribed therapy among older patients was documented in culturally very similar neighbouring population [29], as well as in other populations [30]. On the contrary, almost half of the subjects in US-based study obtained a college degree or higher [18] and they were more likely to manifest critical thinking. Therefore, the small number of subjects achieving a less than maximum score, coupled with the small number of items (3 for TC and 4 for CT subscale), ultimately led to a poor internal consistency for two subscales in this study.

Furthermore, a small increase in the Cronbach’s alpha coefficients can be achieved for PC subscale if items TS11 and TS28 are deleted. The same is observed for the NC subscale if item TS32 is deleted, but we believe that the increase is not significant enough to validate the omission of these items.

Exploration of the floor and ceiling effect showed that high ceiling effect was present in the TC, NC and CT subscales, while the PC subscale had ceiling effect only slightly above the desired value. In a previous study, conducted by Peipert et al. similar high ceiling effects (ranging from 31.6 to 75.9) were observed [18]. This might suggest that the questionnaire is not able to distinguish between those at the higher end of the satisfaction spectrum.

TSC was the only subscale with a very prominent floor effect (62.3%). We believe that that a cause lies in the nature of questions as they estimate satisfaction with explanation how patient’s health and treatment may affect his/her normal work, normal daily activities, personal relationships and emotions. These topics are seldom, if ever discussed in Serbian health care system. Furthermore these questions relate to the communication with treatment staff. In the Serbian health care system, patients interact with only physicians and nurses, therefore, the questions could cause confusion to whom the questions are referring to. In addition, the overall structure of the Serbian healthcare system, along with existing allied healthcare professions differs compared to other healthcare systems, like in the United States. For example, in Serbia treatment staff refers solely to the nurses and physicians involved in patient care, while in the US this may refer to physician assistants, nurse practitioners, medical assistants, pharmacists, and other allied healthcare professionals alongside the patient’s assigned nurse and physician. All of these factors could explain the exceptionally high percentage of subjects that reported a minimal score.

To examine concurrent validity, we used QPS, which is the official questionnaire currently used in Serbia that has also been previously validated [16]. This questionnaire gives two scores which represent patient satisfaction with medical staff and contextual patient dissatisfaction. Given the fact that QPS examines patient satisfaction within a primary health care environment, we believe that it is not the most optimal tool to assess patient satisfaction and treatment satisfaction in chronic diseases, but rather it was the only questionnaire that we had at our disposal. Nevertheless, the FACIT-TS-PS total score, showed a positive correlation with patient satisfaction with medical staff and a negative correlation with contextual patient dissatisfaction. Similar correlations were also observed for the PC and CT subscales.

We found no statistically significant difference between all the subscales and the total score in the test-retest setting, and results correlated well for all subscales except CT. It should be noted; however, that the majority of patients scored maximum points in test and retest for this subscale, without any scores below 10, so therefore there was a small variability in scores and no linear correlation could be observed.

### Limitation

This study was conducted in a single hospital and all subjects were patients with malignant disease. This led to an elderly age of subjects. Furthermore, the study was conducted in a large hospital centre to whom patients of various socio-economic status from the Belgrade area and beyond gravitate to; however, the inclusion of patients with other chronic diseases would have made the study stronger and contributed to less bias.

## Conclusion

This study showed that the Serbian version of FACIT-TS-PS could be used as an instrument to assess patient and treatment satisfaction in chronically ill patients in the Serbian population. We believe that the TSC subscale should be omitted from the questionnaire because it contains questions that are not relevant for patients in Serbia.

## Supporting information

S1 Data (XLSX)
